# Zinc Induced Aβ_16_ Aggregation Modeled by Molecular Dynamics

**DOI:** 10.3390/ijms222212161

**Published:** 2021-11-10

**Authors:** Anna P. Tolstova, Alexander A. Makarov, Alexei A. Adzhubei

**Affiliations:** Engelhardt Institute of Molecular Biology, Russian Academy of Sciences, Vavilov St. 32, 119991 Moscow, Russia; aamakarov@eimb.ru

**Keywords:** Aβ_16_, aggregation, metal binding site, zinc ion, MD, beta-amyloid

## Abstract

It is widely accepted that the addition of zinc leads to the formation of neurotoxic nonfibrillar aggregates of beta-amyloid peptides Aβ_40_ and Aβ_42_ and at the same time destabilizes amyloid fibrils. However, the mechanism of the effect of zinc on beta-amyloid is not fully understood. In this study, a fast zinc-induced aggregation of Aβ_16_ (as compared to a system without zinc) via the formation of Aβ_16_ dimers with one zinc ion coordinated in the metal-binding site _11_EVHH_14_, followed by their polymerization, has been studied by molecular dynamics. The best aggregation was shown by the system composed of Aβ_16_ dimers bound by one zinc ion, with no additional zinc in solution. The presence of Aβ_16_ dimers was a major condition, sufficient for fast aggregation into larger complexes. It has been shown that the addition of zinc to a system with already formed dimers does not substantially affect the characteristics and rate of aggregation. At the same time, an excessive concentration of zinc at the early stages of the formation of conglomerates can negatively affect aggregation, since in systems where zinc ions occupied the _11_EVHH_14_ coordination center and the His6 residue of every Aβ_16_ monomer, the aggregation proceeded more slowly and the resulting complexes were not as large as in the zinc-free Aβ system. Thus, this study has shown that the formation of Aβ_16_ dimers bound through zinc ions at the _11_EVHH_14_ sites of the peptides plays an important role in the formation of neurotoxic non-fibrillar aggregates of beta-amyloid peptide Aβ_16_. The best energetically favorable structure has been obtained for the complex of two Aβ_16_ dimers with two zinc ions.

## 1. Introduction

There is ample evidence showing that the addition of zinc ions into solution with Aβ peptide leads to the formation of neurotoxic nonfibrillar aggregates of beta-amyloid peptides Aβ_40_ and Aβ_42_ [1,2,3,4] and simultaneously destabilizes the amyloid fibrils [3]. Within these aggregates, the fraction of beta sheets is lower than in amyloid fibrils [5,6]. Zinc ions mainly affect the structure of the N-terminus and loop region between two parts of the beta-hairpin positioned between Phe19 и Leu34 at the C-terminus of Aβ_40_ and Aβ_42_ nonfibrillar aggregates [7]. In the presence of zinc, the salt bridge between Asp23 and Lys28 breaks, but in most cases the beta-hairpin itself is not disrupted [8,9].

The formation and structure of neurotoxic nonfibrillar aggregates of beta-amyloid peptide Aβ_40_ and Aβ_42_ in the presence of zinc have not been fully studied. Existing models describe dimers, in rare cases, quadromers of Aβ [5,6,7]. Information on the structure of larger aggregates is scarce and not specific enough. Thus, it is known from NMR data that a zinc ion bound to the Aβ_40_ monomer is ostensibly coordinated by histidines His6, His13, His14 and the N-terminus of the peptide [10]. However, there is no information about the location of the zinc inside the protein aggregates.

Published data on the effects of zinc concentration on the process and the results of the formation of aggregates from Aβ_40_ and Aβ_42_ are not ample. In the majority of studies of beta-amyloid peptide Aβ_40_ and Aβ_42_ aggregation in the presence of zinc both in models and in experiments, the equimolar concentration of zinc and peptides was used [4,8,9,11]. In some cases, zinc concentration equaled half of the beta-amyloid peptide concentration [5,7]. One study also reported that when the concentration of zinc was significantly higher than the concentration of the peptide, the protein aggregates precipitated [2]. However, we did not find any published estimates of the effect of high zinc concentrations on the structure of Aβ peptide conglomerates.

Publications’ data demonstrate a prime importance of the 1–16 domain of Aβ peptide in the interaction with zinc ions [12]. Two additional facts should be noted: (1) in the proposed models of Aβ_40_ and Aβ_42_ with zinc ions in the coordination center, regions 1–16 and 17–42 do not interact with each other and are spatially distant [7,9,11], and (2) Aβ_16_ is present in vivo in humans as an independent species of Aβ peptides [13,14]. These facts indicate that the 1–16 region of the Aβ peptide plays a distinct role in its function in the organism.

In our view, these facts are underestimated, as we found just a few studies of the zinc effect on aggregation of Aβ_16_ [15,16,17,18]. In the study of Istrate et al. [16], DLS and turbidity measurements of Aβ_16_ at 5 mM concentration showed both the aggregation of free peptide, and zinc-induced aggregation. In this study, we have modeled the concentration of Aβ_16_ comparable with the data from Istrate et al. (10–20 mM). Meanwhile, Aβ_16_ is a convenient model for examining the effect of zinc on the formation of nonfibrillar Aβ aggregates. It contains all the residues required for the coordination of zinc ions (Asp1, Glu3, His6, Asp7, Glu11, His13, His14), there are several available NMR equilibrium structures of Aβ_16_ with and without a zinc ion, it is small, and since there is no hydrophobic Aβ_17–42_ C-terminus that can participate in the interaction with zinc ions, this allows us to exclude the variability of conformations associated with the C-terminus of the longer peptides. According to the NMR data on the location of zinc ions in the coordination center of Aβ_16_ [15,16], it can be assumed that the aggregates of Aβ_16_ in the presence of zinc have more pronounced structural patterns compared to aggregates of longer peptides, as most of the peptide is rigidly fixed around the zinc atom [17]. Moreover, the dimers and monomers of Aβ_16_ interacted with zinc ions in different ways [16]. The authors of the study propose that there can be structural transitions between the monomeric and dimeric forms of Aβ_16_, and that the Aβ_16_ dimer forms a nucleus of further oligomerization. On the other hand, there is evidence that Aβ_16_ in the presence of zinc exists mainly in the monomeric form; in order for polymerization to begin, it is necessary to exclude His6 from the coordination center of the peptide [18].

In connection with the above, it appears appropriate and useful to study the aggregation of Aβ_16_ in the presence of zinc applying molecular dynamics (MD) modelling. The aim of the study has been to obtain models of complexes with a large number of peptides, up to 40 molecules, and to check the effect of zinc concentration on the structure of aggregates.

For this purpose, six systems have been created with different relative concentrations of zinc and Aβ. The initial experimentally solved structures used for modeling were the following: free Aβ_16_ monomer without zinc, Aβ_16_ monomer with zinc ions in the coordination center, and Aβ_16_ dimers with one zinc ion in the coordination center of each dimer (listed in Methods). The systems built using these initial Aβ structures have been submitted to MD simulations for 100–200 ns until the equilibrium aggregates were formed.

Molecular dynamics showed substantial differences in the aggregation speed and the size of the resulting aggregates for monomers and dimers of Aβ_16_ with zinc ions in the coordination center as starting conformations.

## 2. Results

Molecular dynamics is a powerful tool for the computer modeling of conformational changes in peptides and peptide complexes on nanosecond time scales. However, this method has its limitations. Thus, MD modeling that would allow one to obtain all possible equilibrium complexes from several Aβ_16_ peptides with an arbitrary number of zinc ions is beyond the capabilities of reasonably accessible computational resources [19]. The probability of a zinc ion from an arbitrary position in solution to enter the metal-binding site of Aβ_16_ within the time scale of tens or even hundreds of nanoseconds is very small. Such a case is not possible to realize in the MD simulations systems modeling in vivo conditions in the intercellular space of the human brain, where zinc concentration is low.

We assume that more mobile zinc ions have sufficient time to come into contact with all available molecules of Aβ_16_ in a diluted Aβ_16_ solution before the aggregation process starts. Therefore, in this study, we have started from the postulate that zinc was already captured by the metal-binding site of Aβ_16_. This is indirectly confirmed by the data of in vitro experiments, which showed that in the presence of zinc, Aβ aggregation proceeded in a different way than in its absence [1].

**Figure 1:**
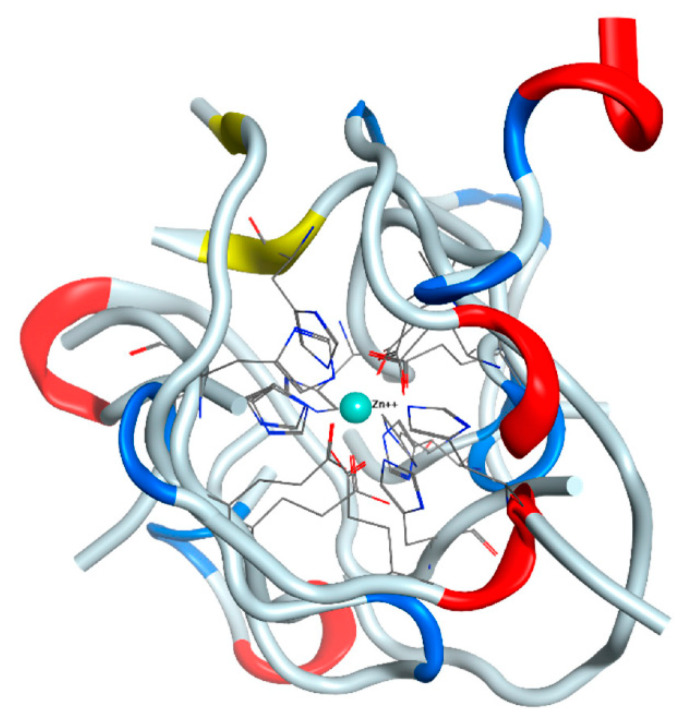
Results of the 100 ns MD simulations of the Aβ_16_ dimer after zinc ion was shifted away from the coordination center. Four final structures are superposed and centered around zinc ion. Zinc coordinating residues are shown.

To assess this scenario and examine how well the chosen force field represents the zinc ion parameters and reflects the experimental data, we have performed the following. The zinc ion from the starting structure of the Aβ_16_ dimer has been removed from its initial position in the coordination center and placed at a distance of 4.6–7 A in such a way that there were no neighboring atoms close enough to capture the ion in the beginning of the MD simulation. We have constructed four such systems (Supplementary Figure S1). In all systems after 100 ns of MD, the zinc ion moved back into the coordination center of the Aβ_16_ dimer and was coordinated by two Glu11 and two His14 residues (Figure 1). In one case, one of the glutamates was replaced by Asp1 from N-terminus of the peptide, in the other, an additional bond was formed with His13. Altogether, we can conclude that the applied MD parameters adequately reproduce the last stages of the zinc binding process.

### 2.1. Aggregation Seeds and Ratios of Zinc and Aβ_16_ Concentration

We have examined two different aggregation seeds as the starting points for the growth of protein conglomerates, i.e., the Aβ_16_ dimer with one zinc ion in the coordination center, and the Aβ_16_ monomer with one zinc ion in the coordination center. Hybrid systems with different ratios of dimers, monomers, and free Aβ_16_ have not been examined in this work.

We have modeled three possible ratios of zinc and Aβ_16_ concentrations.

1. The concentration of zinc ions is higher than the concentration of Aβ_16_, C_zn_ > C_Aβ_. In this case, all Aβ_16_ molecules have one zinc ion at the metal binding site. An example of such a case is the structure PDB:1ZE9, solved by NMR.
2. The concentration of zinc ions is lower than the concentration of Aβ_16_, C_zn_ < C_Aβ_. In this case, part of the Aβ_16_ monomers at early aggregation stages can remain without a zinc ion in the coordination center, and we assumed that in such a system, dimers of Aβ_16_ with one zinc ion coordinating the interaction between peptides could be formed. An example of such a case is the structure PDB: 2MGT, solved by NMR.
3. There are no zinc ions in the system, C_zn_ = 0. An example of such a case is the structure PDB:1ZE7, solved by NMR.

### 2.2. MD Modeling

Three systems representing these conditions were constructed. The molecules in each system were placed in the cubic water box with the edge of 15 nm and located at a sufficient distance from each other so that there was no need to take into account the effect of the initial arrangement of molecules on the resulting complexes. This led to restrictions on the number of Aβ_16_ molecules in the water box.

1. C_zn_ > C_aβ_. System based on the PDB:1ZE9 structure. The system was created comprising 1 Aβ_16_ dimer with a zinc ion as a polymerization seed, and 19 Aβ_16_ monomers with a zinc ion in the coordination center (System 1 in Table 1).
2. C_zn_ < C_Aβ_. System based on the PDB:2MGT structure. The system was created comprising 19 Aβ_16_ dimers bound by a zinc ion in the coordination center (System 2 in Table 1).
3. C_zn_ = 0. System based on the PDB:1ZE7 structure. The system was created comprising 20 Aβ_16_ monomers without zinc (System 3 in Table 1).

In all the above systems, the molecules were located at a sufficient distance from each other so that there was no need to take into account the effect of the initial arrangement of molecules on the resulting complexes. The systems have been submitted to MD simulations. After 100 ns simulation, protein conglomerates were formed in all three systems.

In the first case (C_zn_ > C_aβ_) in System 1 from Table 1, after 100 ns of MD, a complex of 6 Aβ_16_ molecules, two complexes of three Aβ_16_ molecules, and one complex of 4 Aβ_16_ molecules were formed. Four Aβ_16_ molecules remained in monomeric form. The connectivity length was C_L_ = 11.91.

In the second case (C_zn_ < C_aβ_) in System 2 from Table 1, after 100 ns of MD, one complex of 8 Aβ_16_ dimers, one complex of 7 Aβ_16_ dimers, and two complexes of two dimers were formed. The last two complexes were located very close to the second conglomerate, so this cluster could be identified as a single complex of 11 Aβ_16_ dimers. The connectivity length for this system was C_L_ = 6.15. An additional MD simulation of 100 ns was carried out for this system, which showed that upon reaching the time of 200 ns, one large complex was formed in the system containing all Aβ_16_ molecules (Figure S7).

In the third case (C_zn_ = 0) in System 2 from Table 1, after 100 ns of MD, one complex of 10 Aβ_16_ molecules and 4 Aβ_16_ dimers were formed. Two Aβ_16_ molecules stayed in monomeric form. The connectivity length was C_L_ = 8.82.

**Figure 2:**
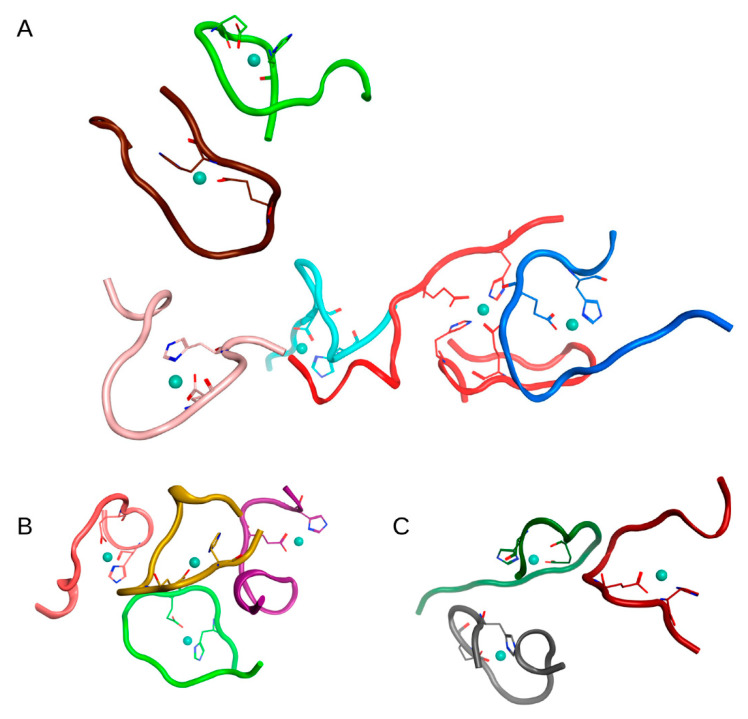
Results of 100 ns MD simulation of the Aβ_16_ System 1 from Table 1 (C_zn_ > C_Aβ_). Three large clusters are shown (**A**–**C**). Box size is 15 nm. The Aβ_16_ molecules are shown with different colors. Zinc coordinating side chains are shown. Zinc ions are highlighted in blue. The Aβ_16_ dimer (bottom right corner in **A**) is colored red.

**Figure 3:**
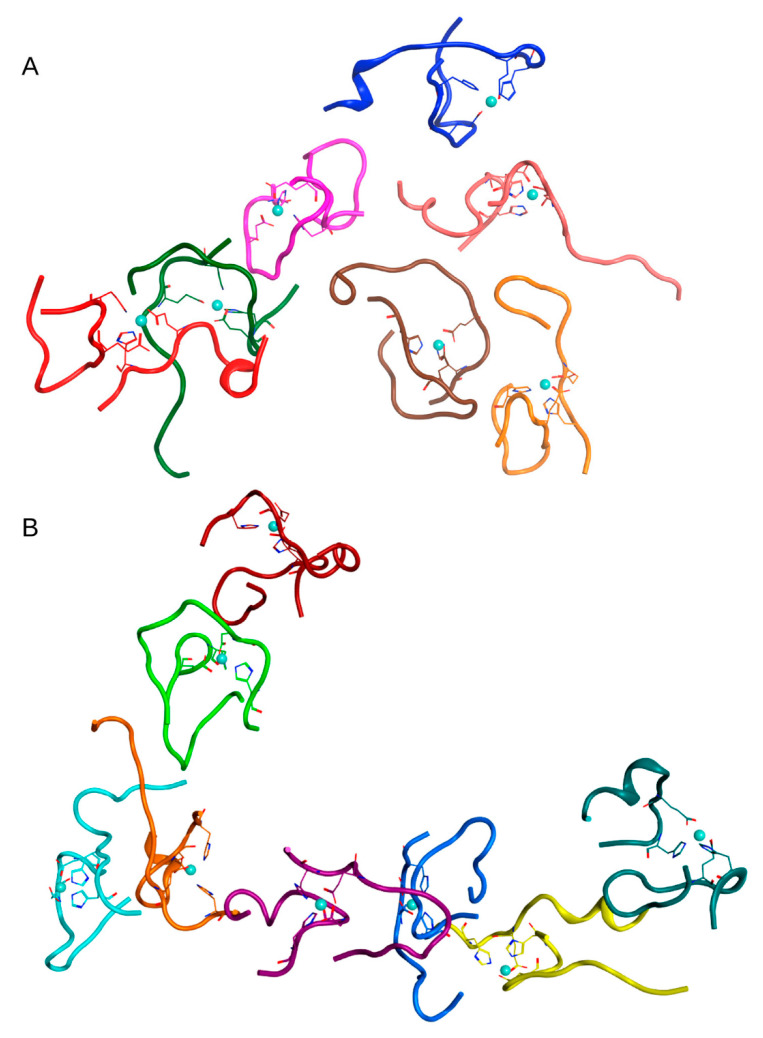
Results of 100 ns MD simulation of the Aβ_16_ System 2 from Table 1 (C_zn_ < C_Aβ_). Two large clusters are shown (**A**), (**B**) Box size is 15 nm. The Aβ_16_ molecules are shown with different colors. Zinc coordinating side chains are shown. Zinc ions are highlighted in blue.

**Figure 4:**
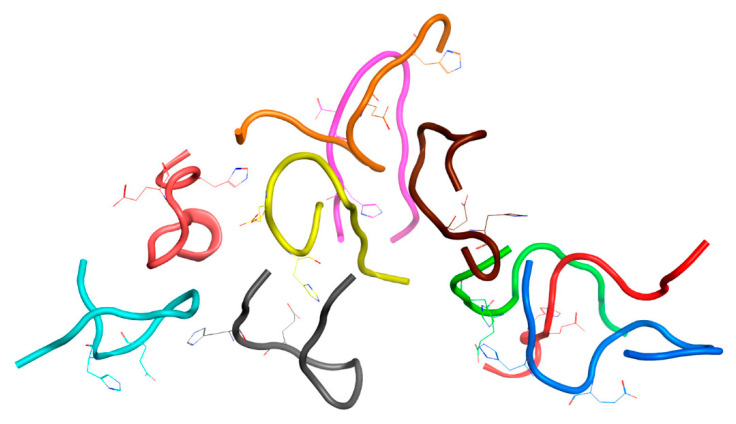
Results of 100 ns MD simulation of the Aβ_16_ System 3 from Table 1 (C_zn_ = 0). The largest cluster is shown. Box size is 15 nm. The Aβ_16_ molecules are shown with different colors. Zinc coordinating side chains are shown. There is no zinc in this system.

The MD results for these systems are shown in Figures S2–S4 and major complexes from each system are shown in Figure 2, Figure 3 and Figure 4 and described in Table 2. An unexpected result was that the difference in connectivity length C_L_ between Systems 1 and 2 was almost 50%.

### 2.3. MD Modeling of the Systems Created to Probe the Main Results

**Figure 5:**
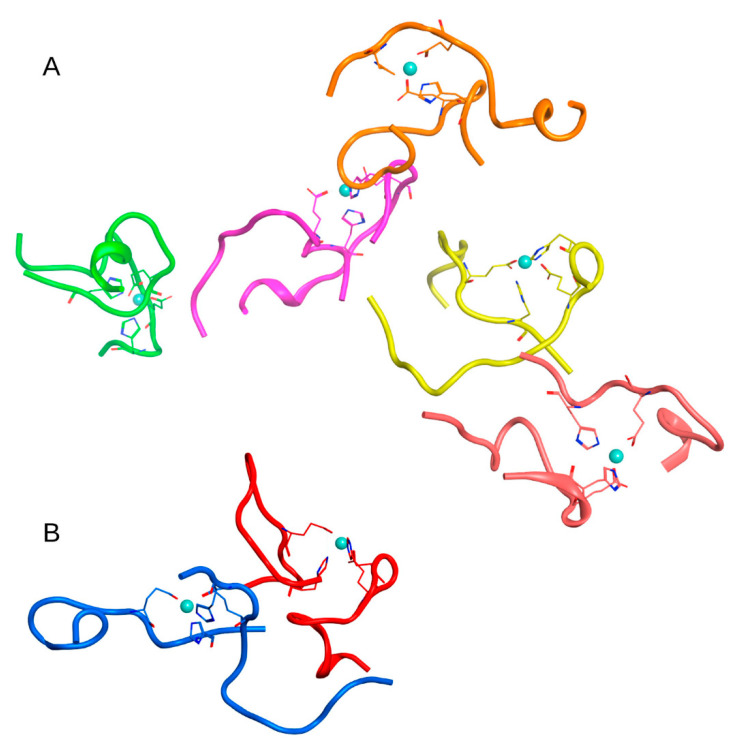
Results of 100 ns MD simulation of the Aβ_16_ System 4 from Table 1 (C_zn_ < C_Aβ_). Two large clusters are shown (**A**,**B**). Box size is 15 nm. The Aβ_16_ molecules are shown with different colors. Zinc coordinating side chains are shown. Zinc ions are highlighted in blue.

To exclude the effect of high initial concentration of Aβ_16_ dimers on the results, we have created a system of nine dimers in a cell of the same dimensions as before, with a minimum distance between molecules > 4.1 nm, thus reducing the concentration of Aβ_16_ by half (System 4 in Table 1). After 100 ns of MD, conglomerates were also formed in this system, comprising one complex of five dimers and one of two dimers (Figure 5 and Figure S5). Two other dimers remained outside of the aggregation conglomerates. Nevertheless, this is a rather high aggregation result for such a diluted system with a large initial distance between molecules.

The questions arise, how does the process of aggregation proceed in a solution when the concentration of zinc is higher than the concentration of Aβ_16_? Does the concentration of zinc affect this process and the pattern of conglomerates? If monomers of Aβ_16_ complexed with zinc ions are formed almost instantly, then further aggregation is likely to proceed slowly and, possibly, large clusters will not be formed. If dimers are formed first, how will additional free zinc ions affect the character and rate of aggregation?

**Figure 6:**
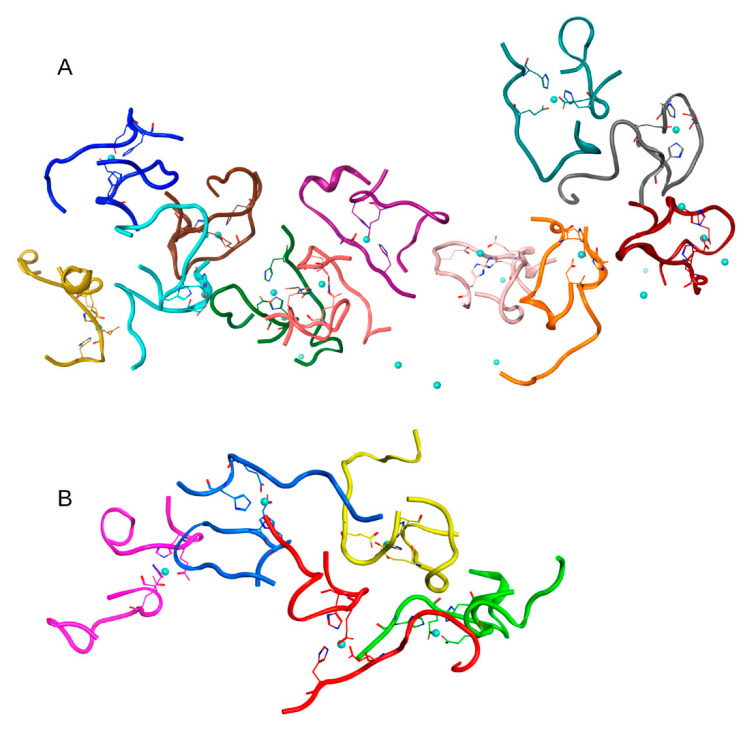
Results of 100 ns MD simulation of the Aβ_16_ System 5 from Table 1 (C_zn_ > C_Aβ_). Two large clusters are shown (**A**), (**B**) Box size is 15 nm. The Aβ_16_ molecules are shown with different colors. Zinc coordinating side chains are shown. Zinc ions are highlighted in blue.

**Figure 7:**
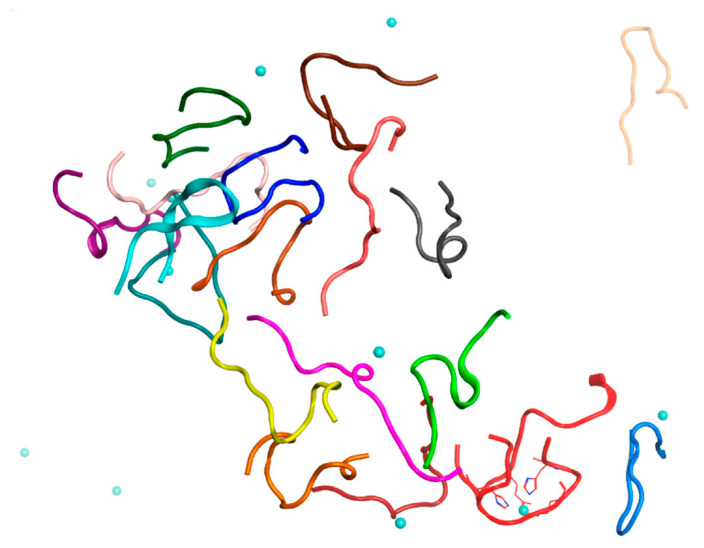
Results of 150 ns MD simulation of the Aβ_16_ System 6 from Table 1 (C_zn_ > C_Aβ_). Box size is 15 nm. The Aβ_16_ molecules are shown with different colors. Zinc coordinating side chains are shown. Zinc ions are highlighted in blue. The Aβ_16_ dimer is colored red.

To answer these questions, two systems were created. One was based on the above System 2 from Table 1; in this system, 20 zinc ions were randomly added to the solution (System 5 in Table 1). The other system consisted of one Aβ_16_ dimer with zinc, 19 Aβ_16_ molecules without zinc and 20 zinc ions randomly located in a solution (System 6 in Table 1). The MD simulation results for these systems are shown in Figure 6 and Figure 7 and Figure S6. In both systems after 100 ns of MD, large clusters were formed, combined with single free Aβ_16_ molecules.

MD modeling has shown that the addition of zinc to a system where dimers have already been formed does not affect polymerization. Connectivity length after 100 ns of MD for the system with 19 dimers and 20 additional zinc ions (System 5 in Table 1) was C_L_ = 6.12, which is comparable to C_L_ = 6.15 for a similar system 2 from Table 1 where there were no additional free zinc ions.

MD modeling of System 6 from Table 1 showed that although single free zinc ions were located near the protein complexes, they did not form hydrogen bonds with Aβ_16_. Perhaps this was due to the limitations of molecular dynamics method in general and the force field used for zinc ions in particular. However, the force field used in our MD modeling increases the contribution of electrostatic interactions and should facilitate rapid aggregation.

On the whole for both systems with additional free zinc, although it is hard to study the incorporation of additional zinc ions from solution into the coordination centers of peptides on the molecular dynamics time scale, we can say that this is a much longer process than aggregation. It is quite probable that the presence of free zinc ions will not affect further aggregation of the already formed complexes as strongly as in the case when zinc initially occupied all possible metal-binding sites of each peptide. This conclusion underlines the important role of Aβ_16_ dimerization around a single zinc ion in the aggregation process. The MD modeling results obtained in this study are summarized in Table 2.

### 2.4. Clustering and Identification of Preferable Aβ_16_ Dimer Structure by REMD Simulation

We have performed structure comparison and clustering of the Aβ_16_ dimer conformations from the MD simulation results to identify a preferable structure. A total number of 28 dimers from two systems were clustered, 19 dimers after 200 ns MD (System 2 Table 1) and 9 dimers after 100 ns MD (System 4 Table 1). In this subset 26 dimers were part of larger complexes. The structural alignment of all dimers showed an RMSD difference of 5.54 Å. The MaxCluster program was used for the clustering of dimer structures. As a result of clustering with different settings, a cluster with a maximum number of molecules (9 out of 28) has been obtained using the nearest neighbor clustering method. This cluster had a high threshold RMSD value of 5 A, which did not allow a representative structure to be identified directly from these results. At the same time the remaining dimers were not included in clusters. Other clustering methods or a lower RMSD cut-off resulted in smaller final cluster size or no clusters at all. This result was expected, since Aβ peptides are highly mobile in solution, and experimental methods did not show the presence of a clear structure in the resulting complexes of Aβ with zinc [20,21].

**Figure 8:**
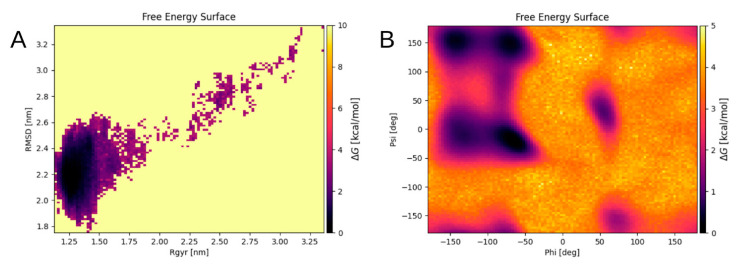
Energy landscape of the Aβ_16_ quadromer with two zinc ions in the coordination centers of dimers after 100 ns of REMD simulation with 32 replicas, in the temperature range of 300–382.84 K. (**A**) Gibbs free energy surface as a function of the RMSD value and the gyration radius of the Aβ_16_ conglomerate and (**B**) of the values of phi and psi angles for Aβ_16_ molecule.

Nevertheless, we have succeeded in identifying a preferred structure for the system of two dimers coordinated by two zinc ions. For this purpose, a replica exchange molecular dynamics (REMD) simulation of the structure of two Aβ_16_ dimers has been carried out to obtain a complete ensemble of possible conformations of this system, and to select energetically favorable conformations. As the initial structure, a random compact structure was chosen from the resulting globule of 19 dimers (System 2 Table 2) after 200 ns of MD. The resulting energy landscape of the final system was obtained as a graph of the free energy changes (Figure 8). The graph was calculated as a function of the RMSD and the radius of gyration of the Cα atoms, and of the Phi and Psi angles of the system. Each point on the graph corresponds to a specific conformation of the quadromer. Dark areas show local minima corresponding to the most energetically favorable structures.

**Figure 9:**
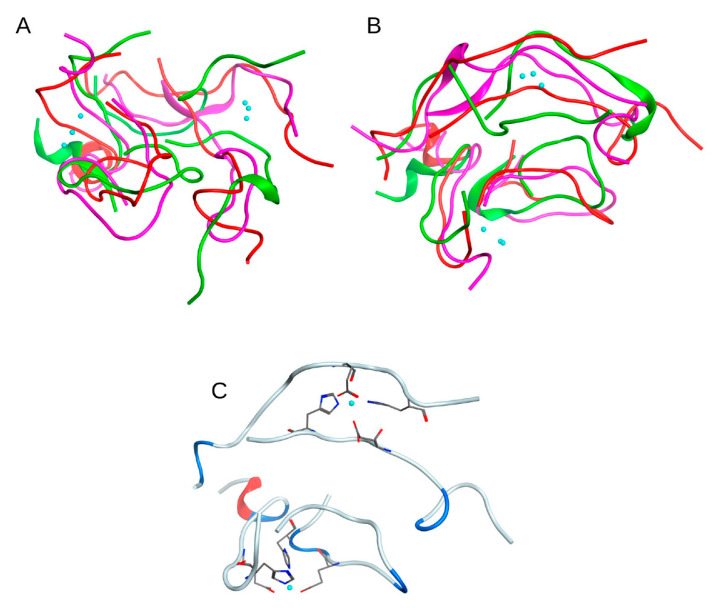
Three conformations with the lowest free energy according to the analysis of energy landscapes from REMD modeling of a complex of four Aβ_16_ molecules with two zinc ions in the coordination centers of Aβ_16_ dimers. (**A**,**B**) represent different views of the superposition of the three complex conformations. For clarity, each complex structure formed by four Aβ_16_ molecules is highlighted in the same color. Zinc ions are shown in blue. (**C**) shows one of the three conformations. Zinc coordinating side chains are shown.

The conformations from the local minima of these two landscapes (Figure 8A,B) intersect, three conformations correspond to different minima of the graph shown in Figure 8B and to the only minimum of the graph in Figure 8A. These conformations were selected as representative. They appeared at a long distance in time on an MD scale and were formed separately from each other. Although these structures were not identical, they were still quite similar, showing generally the same packing pattern (Figure 9A,B). This is supported by the fact that the zinc ions in these structures are located very close to each other. For the quadromer structure shown in Figure 9C, chemical shifts for Hα atoms of each residue were calculated with SPARTA+ server [22] and compared with the NMR data of two different Aβ_16_ dimer isoforms. The results are shown in Figure S8. They show a close similarity of the REMD and NMR data and the convergence of REMD simulation.

## 3. Discussion

As we have observed earlier, Aβ_16_ represents a good model for examining the zinc effects on the aggregation process of different types of Aβ peptide. In all Aβ variants the segment 1–16 is responsible for electrostatic interactions, whereas segment 17–42 participates in hydrophobic interactions. There is no doubt that the C-termini of Aβ_42_ and Aβ_40_ peptides form intermolecular bonds in protein conglomerates, but only the N-terminus is zinc sensitive. Moreover, a number of studies reported the resilience of the structural patterns of the C-terminus against minor changes in the relative location of several residues in the presence of zinc ions [8,12].

Although we cannot extrapolate the data obtained for Aβ_16_ directly to Aβ_42_ and Aβ_40_ peptides, interaction inside Aβ_16_ dimers occurs via the _11_EVHH_14_ site and does not involve C-terminal residues. We can therefore hypothesize that this interaction involves the same sites in longer peptides and that the structural data obtained in this study is useful for Aβ_42_ and Aβ_40_ nonfibrillar aggregates with zinc.

In the previously cited paper of Istrate et al. [16], the aggregation of free Aβ_16_ peptide and zinc-induced aggregation in the presence of a two-fold molar excess of ZnCl_2_ was shown by DLS and turbidity technique for the peptide at 5 mM concentration. A significant increase of turbidity for Aβ_16_ solution in the presence of a two-old molar excess of ZnCl_2_ was observed in contrast with the free peptide solution. In Table 1 of the paper, the mean diameter of oligomers of Aβ_16_ was shown as 2.5 ± 0.3 nm. In comparison with the mean dimer diameter of 1.8 ± 0.7 nm calculated by us for the NMR PDB:2MGT structure, the size of oligomers in the Istrate et al. study is not large, about 3–4 Aβ_16_ molecules per aggregate. In our study, we aimed to explore conglomerates of various sizes and surmised that larger conglomerates can appear at higher concentrations of Aβ_16_. We have accordingly increased the concentration of Aβ_16_ in our MD systems to 10 mM, which is still within the range of the Istrate et.al. data.

Concerning the zinc location in the Aβ_16_ aggregates, it should be stated that a choice of particular force field affects the results of the MD modeling dramatically. A calculation of accurate and proper general parameters for metal ions in metalloproteins is a big challenge that has not yet been resolved. The main problem is that the metal ion polarizes the neighboring residues, leading to partial charge redistribution. Quantum mechanics calculations conducted for metalloproteins show a noninteger partial charge on metal ion and on the neighboring residues [11]. Partial charges on atoms of flexible molecules are constantly changing in a real environment, depending on a current reciprocal arrangement of atoms. However, there is no option to change the partial charges during MD simulation. In addition, noninteger charges can lead to artifacts and errors in the MD modeling if a charged group of atoms, which acquires a combined integer charge, is disrupted when a metal ion changes its location and moves outside of this group.

In this study, we have used integer charges without polarization on zinc ions and with redistribution of partial charges on every residue suitable for zinc coordination. This leads to a possibility for the zinc ion to move in the solution and change coordination center without problems with partial charges. However, as the downside of this approach, ion bridges that form between zinc ions and the coordinating atoms in MD modeling are perhaps excessively strong, and there were no cases when these interactions were broken in our simulations, though there were no constraints imposed on zinc ion location changes.

There is evidence that in vitro a conversion can occur between monomeric and dimeric forms of Aβ_16_ [18], but in our model such transitions were not implemented. Therefore, we have built two different systems with monomeric and dimeric Aβ_16_ to explore differences between these states.

A surprising result was that the monomeric form of Aβ_16_ with zinc ions in its coordination center aggregates even to a lesser extent than Aβ_16_ without zinc. This fact can be explained by the difference between monomeric and dimeric forms of Aβ_16_ with zinc ions bound to the _11_EVHH_14_ site. The monomer has zinc coordinated by residues His6, Glu11, His13 and His14, whereas in the dimeric form, the zinc ion is coordinated by Glu11 and His14. There is data showing that the _11_EVHH_14_ site plays a crucial role in zinc-induced Aβ peptide aggregation [16,17,18], and that H6R mutation facilitates zinc-induced dimerization of the Aβ_16_ [23]. This fact is also confirmed by a substantially higher level of aggregation of the Aβ_6–16_ compared to the Aβ_16_ [16].

Such a substantial difference in the aggregation of dimeric and monomeric forms of Aβ_16_ with zinc ions in the coordination center indicates that, firstly, the aggregation of Aβ_16_ in the presence of zinc occurs most likely through an intermediate stage of Aβ_16_ dimerization involving one zinc ion, and secondly that an excessive concentration of zinc can, in contrast with low and medium zinc concentrations slow down the aggregation of Aβ_16_.

As has been noted earlier, Aβ_16_ is a rather short peptide, and the zinc ion anchors and makes more rigid a sizeable segment of it, making it possible to uncover patterns of the Aβ_16_ peptide folding into aggregates. Analysis of the REMD modeling trajectories showed that all energetically favorable conformations of the Aβ_16_ quadromer structure acquired from the energy minima demonstrate the same packing and interaction patterns. They may represent snapshots of the same conformation, with slightly changing relative positions of peptide chains, within the duration of MD simulation time. However, with a longer peptide, things may be not so simple because of the flexible C-terminus. A study of Aβ_42_ oligomerization in complex with zinc ions performed along the lines of how it has been done for Aβ_16_ in this work can provide new insights into the zinc-induced Aβ aggregation mechanism and specifically into the role of the C-terminus in the aggregates, especially in comparison with the data presented here. We therefore aim to develop an alternative approach to identify the preferred conformations of Aβ_42_ complex with zinc, since the REMD method is computationally not feasible for such a system.

## 4. Materials and Methods

The initial structures of the amyloid beta peptide were obtained from PDB bank of protein structures: PDB:2MGT [16] PDB:1ZE9 [15] and PDB:1ZE7 [15]. These are NMR structures of the Aβ_16_ dimer and monomer with a zinc ion coordinated in the metal-binding site _11_EVHH_14_, and the Aβ_16_ monomer, respectively. PDB structure 2MGT contained the English mutation H6R, so in the original structure PDB:2MGT Arg6 has been replaced by His6 in each Aβ_16_ molecule to obtain unmodified peptides.

These structures were placed in cubic cells with a box size of 15 nm in such a way that the minimum distance between molecules was greater than the double cutoff radius of van der Waals forces (2.4 nm), water and NaCl ions at a concentration of 115 mM were added, and the system was simulated by molecular dynamics for 100 ns utilizing the GROMACS software package [24].

The following molecular dynamics protocol was used for all systems. MD simulations (structure relaxation) were carried out with the GROMACS software [24]. All models were first processed by energy minimization procedure sequentially using the steepest descent algorithm, and then conjugated gradients until a local minimum was reached. Then a two-stage equilibration of the system was carried out in NVT (the number of particles, volume and temperature were constant) and NPT (the number of particles, pressure and temperature were constant) ensembles for 100 ps, respectively. In the simulation, the Ewald summation algorithm was used, the constraints on the motion of atoms were set using the LINCS algorithm. The cutoff radii of the Coulomb and Van der Waals potentials were 1.2 nm. The time step was 0.2 fs. All systems included periodic boundary conditions. Water and ions were modeled explicitly using the TIP3 model for water.

For adequate modeling of the peptide interactions with zinc ions, a specially developed force field was chosen [25]. For structural clustering, we used the program MaxCluster (Available online: http://www.sbg.bio.ic.ac.uk/maxcluster/index.html, accessed on 2 August 2021) As a quantitative parameter to estimate the degree of aggregation in different systems with Aβ_16_, we used connectivity length [26] $L_{c}=\sum_{i}\sqrt{N_{i}}$, a parameter equal to the sum of square roots of the number of molecules N in each complex of the system, over all i complexes formed in the system. Thus, the smaller the connectivity length, the stronger the aggregation. Each dimer in this calculation was counted as one molecule.

In this study, a replica exchange molecular dynamics simulation (REMD) has been performed of a system with two Aβ_16_ dimers coordinated by two zinc atoms in a water-salt solution with a concentration of 115 mM NaCl ions. We used 32 replicas with a temperature range from 300.0 to 382.84 K. The simulation time was 105 ns for each replica, 3.2 μs with trajectory recording in total. The replica rate was 11% with a replex value of 2000.

## 5. Conclusions

In this study we have demonstrated that the basic structure of a zinc-coordinated Aβ_16_ dimer is the nucleus of aggregation in unstructured clusters. The presence or absence of additional free zinc in solution has practically no effect on further polymerization, if the dimers have already been formed. However, if Aβ_16_ exists as a monomer incorporating a coordinated zinc ion, the aggregation proceeds at such a slow rate that it is beyond the scope of modeling by the molecular dynamics method.

## Figures and Tables

**Table 1:** Description and MD simulation parameters for all systems modeled in this study, with various Aβ_16_ initial structures and zinc concentrations.

N	System Description	Minimal Distance between Molecules, nm	MD Simulation Time, ns
1	1 dimer of Aβ_16_ with zinc ion in coordination center based on PDB:2MGT + 19 monomers of Aβ_16_ with zinc ion in coordination center from PDB:1ZE9 + 115 mM of NaCl + water	3.1	100
2	19 dimers of Aβ_16_ with zinc ion in coordination center based on PDB:2MGT + 115 mM of NaCl + water	2.4	200
3	20 monomers of Aβ_16_ without zinc based on PDB:1ZE7 + 115 mM of NaCl + water	2.7	100
4	9 dimers of Aβ_16_ with zinc ion in coordination center based on PDB:2MGT + 115 mM of NaCl + water	4.1	100
5	19 dimers of Aβ_16_ with zinc ion in coordination center based on PDB:2MGT + 20 zinc ions + 115 mM of NaCl + water	2.4	100
6	1 dimer of Aβ_16_ with zinc ion in coordination center based on PDB:2MGT + 19 monomers of Aβ_16_ without zinc based on PDB:1ZE7 + 20 zinc ions+ 115 mM of NaCl + water	3.1	150

**Table 2:** MD simulation results for the systems from Table 1 containing Aβ_16_ with various zinc concentrations.

N	System Description	Simulation Time, ns	MD Results	Connectivity Length $L_{c}=\sum_{i}\sqrt{N_{i}}$, Where *i* Is the Number of Complexes in the System, *N* Is the Number of Molecules in One Complex
1	1 dimer of Aβ_16_ with zinc ion in coordination center based on PDB:2MGT+ 19 monomers of Aβ_16_ with zinc ion in coordination center based on PDB:1ZE9 + 115 mM of NaCl + water	100	Several small Aβ_16_ aggregates were formed. 4 Aβ_16_ molecules remained in monomeric form.	11.91
2	19 dimers of Aβ_16_ with zinc ion in coordination center based on PDB:2MGT + 115 mM of NaCl + water	200	Fast aggregation into large clusters that merge into single cluster.	6.15 (100 ns) 4.36 (200 ns)
3	20 monomers of Aβ_16_ without zinc based on PDB:1ZE7 + 115 mM of NaCl + water	100	One large cluster of monomers and several small clusters were formed.	10.82
4	9 dimers of Aβ_16_ with zinc ion in coordination center based on PDB:2MGT + 115 mM of NaCl + water	100	Fast aggregation into one cluster, several dimers remained outside of the aggregation conglomerates.	5.65 *
5	19 dimers of Aβ_16_ with zinc ion in coordination center, based on PDB:2MGT + 20 zinc ions + 115 mM of NaCl + water	100	Fast aggregation into one large cluster, two dimers remained outside of the aggregation conglomerates.	6.12
6	1 dimer of Aβ_16_ with zinc ion in coordination center based on PDB:2MGT + 19 monomers of Aβ_16_ without zinc based on PDB:1ZE7 + 20 zinc ions+ 115 mM of NaCl + water	150	One large cluster of monomers, one small cluster with a dimer, and one free Aβ_16_ monomer. Zinc ions float freely in solution and are not found in protein conglomerates.	6.73

* This value was obtained for 9 dimers; hence it cannot be compared with connectivity length values calculated for other systems due to difference in the total number of molecules. The connectivity length is not a normalized parameter.

## Data Availability

Data are available upon request.

## Supplementary Materials

The following are available online at https://www.mdpi.com/article/10.3390/ijms222212161/s1.
