# Supplementary material for: Zinc Induced Aβ16 Aggregation Modeled by Molecular Dynamics
Source: Int J Mol Sci. 2021 Nov 10;22(22):12161. doi: 10.3390/ijms222212161 (PMC8622866; doi:10.3390/ijms222212161)
Supplement: Supplementary file 1 [file ijms-22-12161-s001.zip › ijms-1402213-supplementary.pdf]

International Journal of
Molecular Sciences

Supplementary Materials

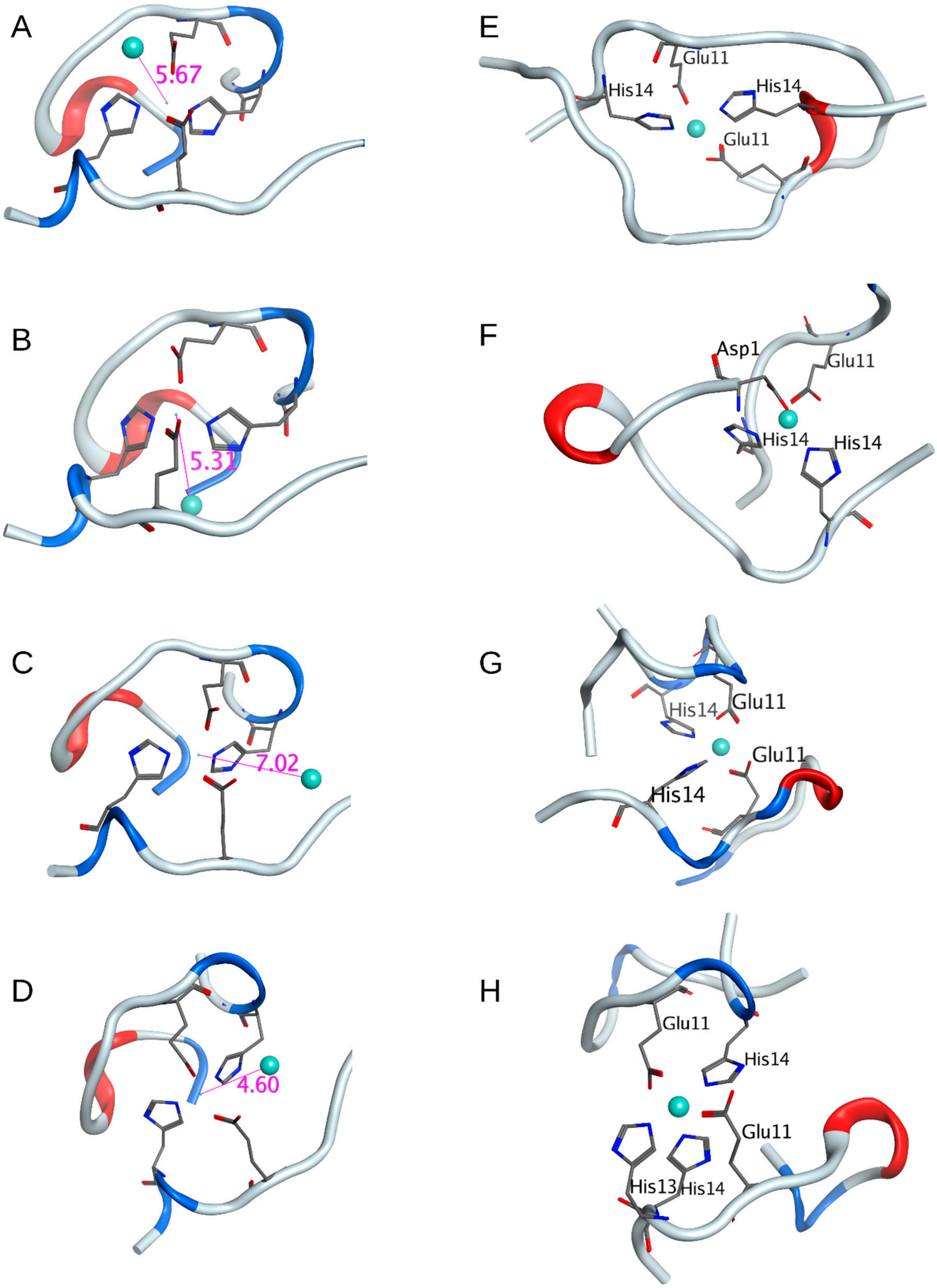

Figure S1. Results of four 100 ns MD simulations of the Aβ_{16} dimer with zinc ion shifted from the coordination center to the four different positions. (**A–D**) initial structures where the distance between the initial point and shifted zinc ion position is shown. The zinc coordinating Glu11 and His14 residues are shown. (**E–H**) final structures after 100 ns MD. Zinc coordinating residues are shown.

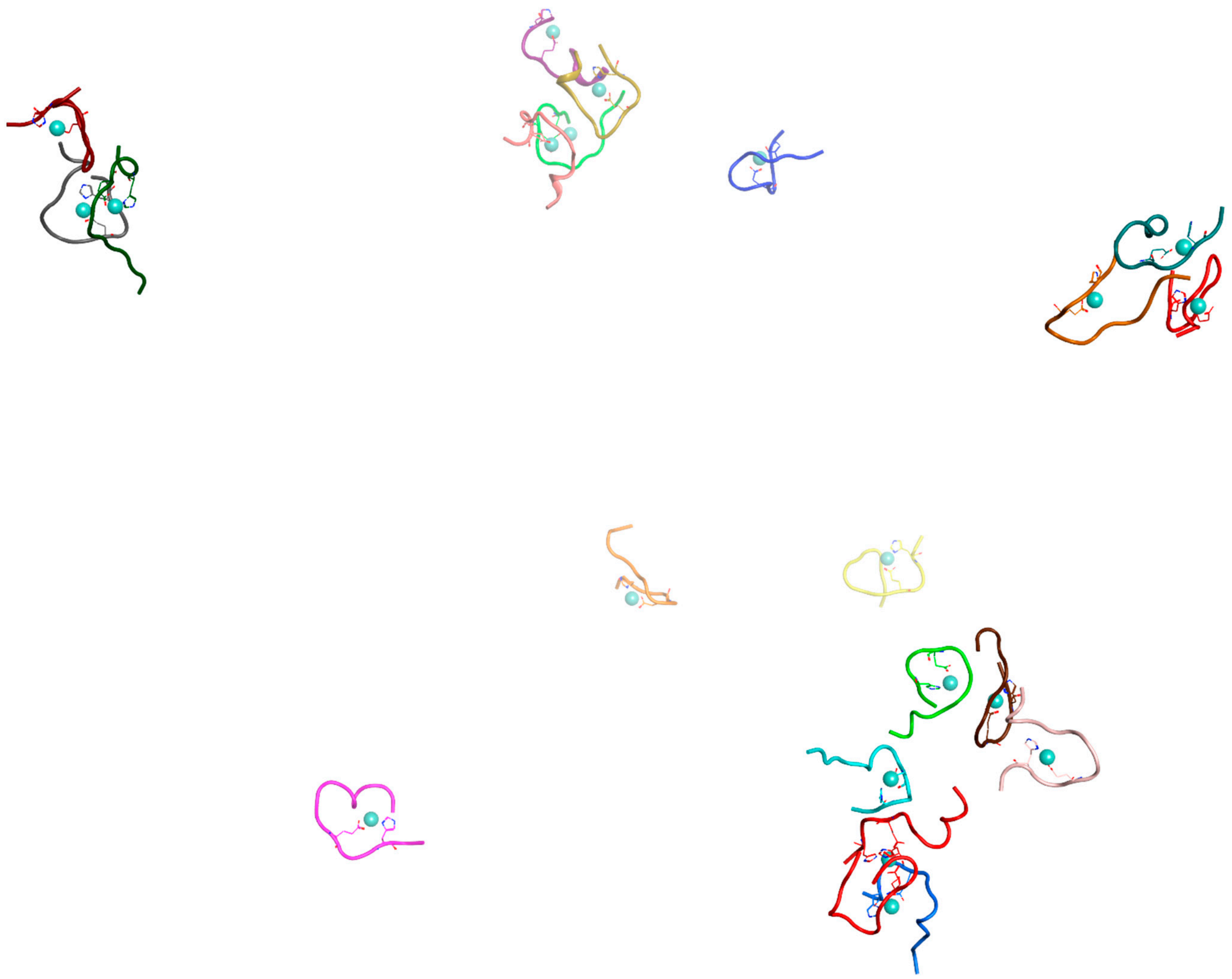

Figure S2. Results of 100 ns MD simulation of the $A\beta_{16}$ System 1 from Table 1 ($C_{zn} > C_{A\beta}$). The $A\beta_{16}$ molecules are shown with different colors. Zinc coordinating side chains are shown. Zinc ions are highlighted in blue. The $A\beta_{16}$ dimer (bottom right corner) is colored red.

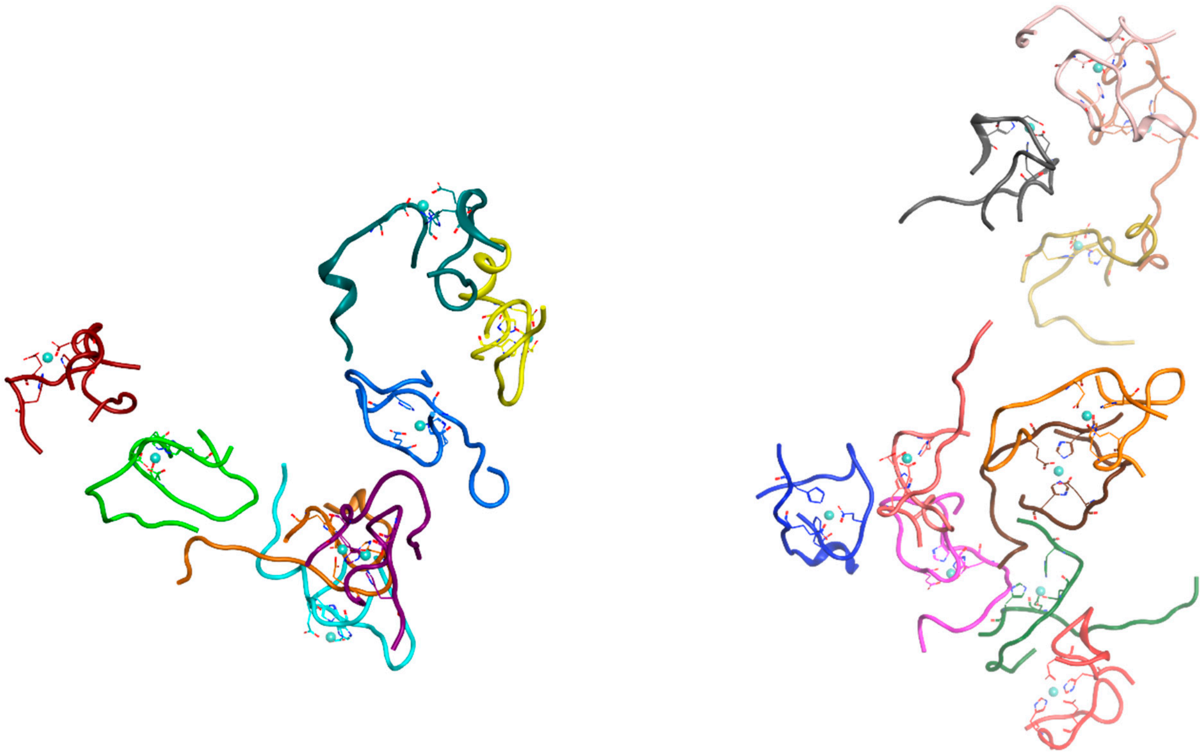

Figure S3. Results of 100 ns MD simulation of the $A\beta_{16}$ System 2 from Table 1 ($C_{Zn} < C_{A\beta}$). Box size is 15 nm. The $A\beta_{16}$ molecules are shown with different colors. Zinc coordinating side chains are shown. Zinc ions are highlighted in blue.

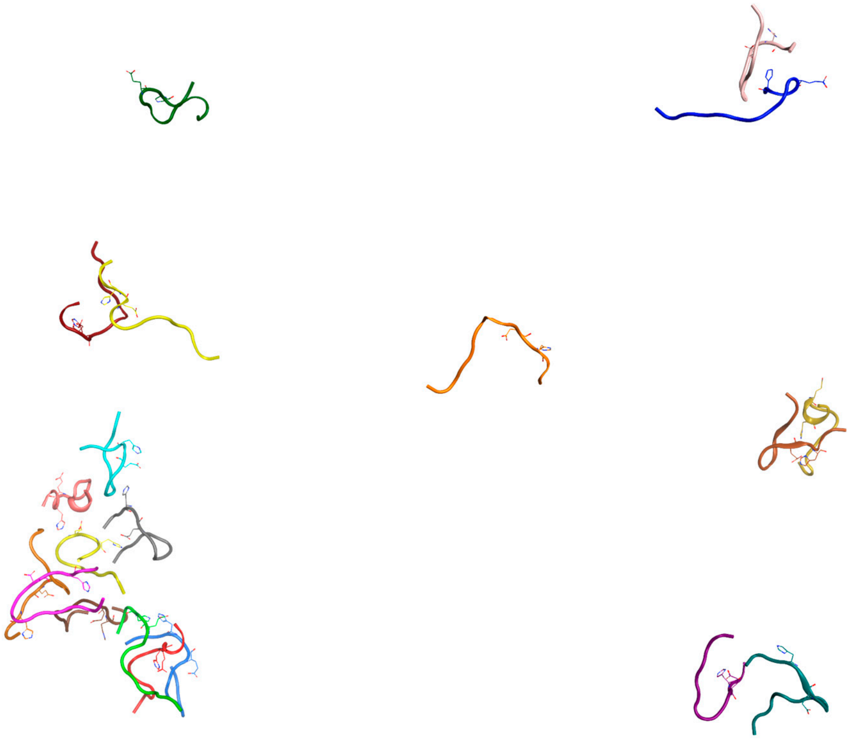

Figure S4. Results of 100 ns MD simulation of the $A\beta_{16}$ System 3 from Table 1 ($C_{Zn} = 0$). Box size is 15 nm. The $A\beta_{16}$ molecules are shown with different colors. Zinc coordinating side chains are shown. There is no zinc in this system.

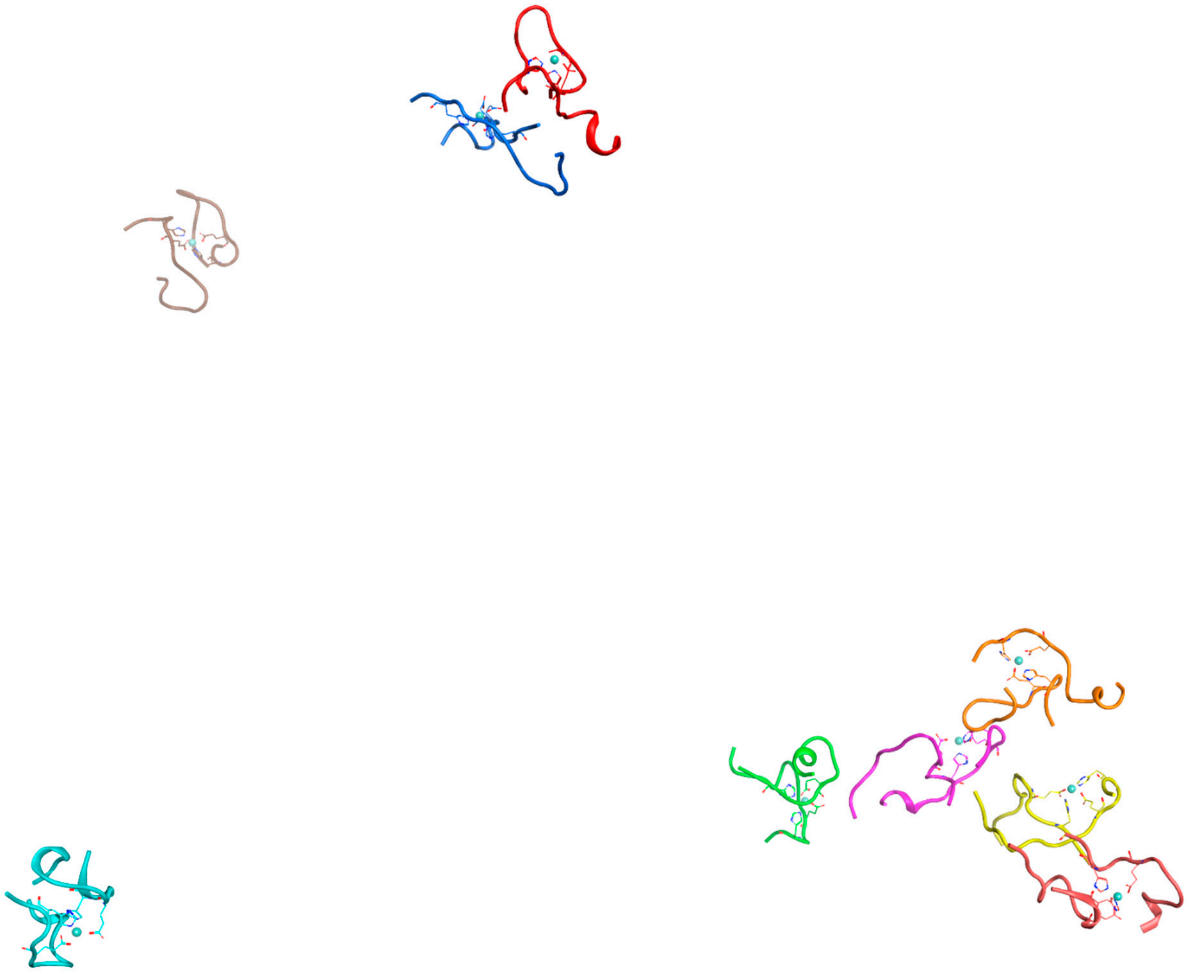

Figure S5. Results of 100 ns MD simulation of the Aβ16 System 4 from Table 1 (C_{Zn} < $C_{A\beta}$). Box size is 15 nm. The Aβ16 molecules are shown with different colors. Zinc coordinating side chains are shown. Zinc ions are highlighted in blue.

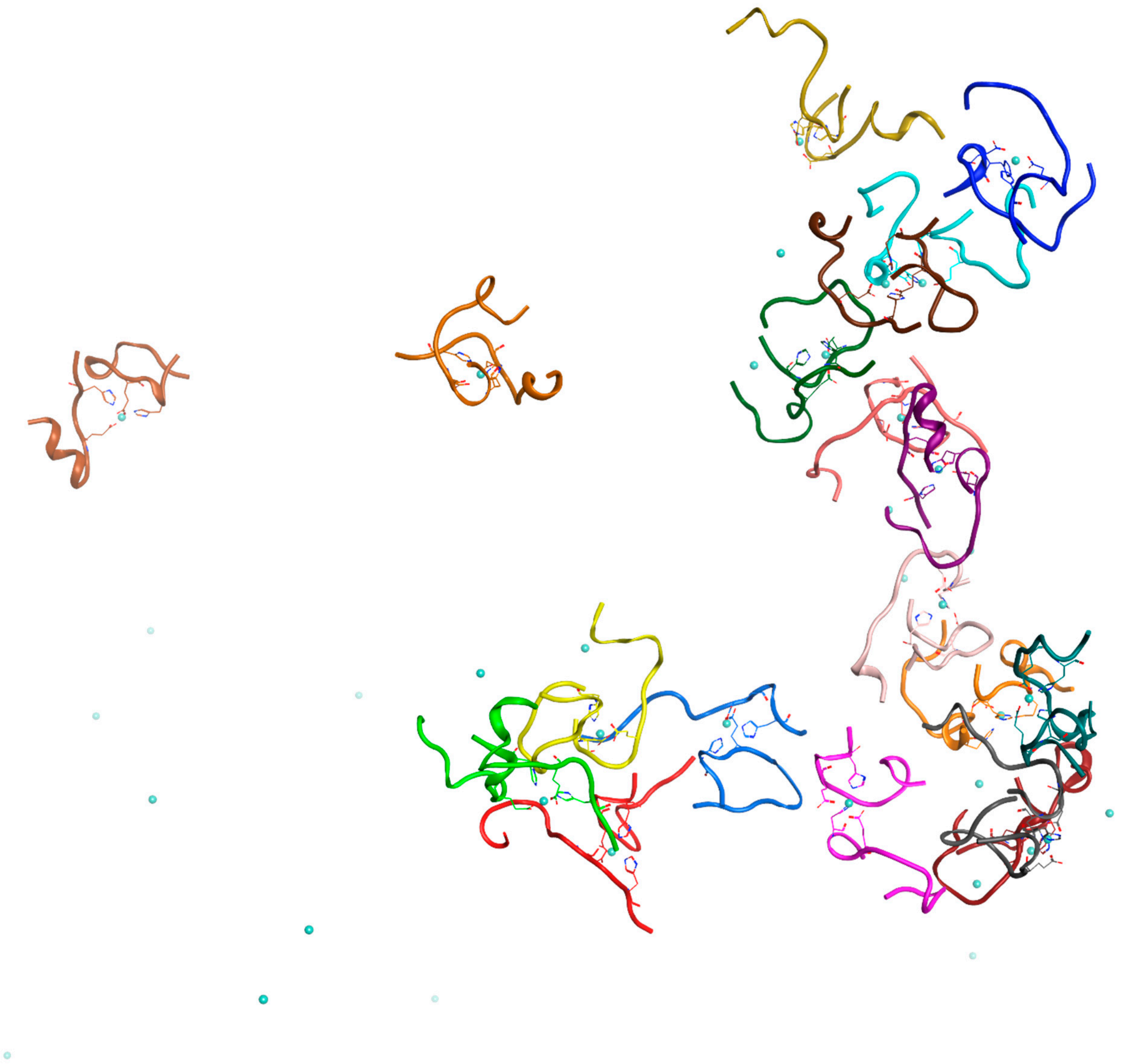

Figure S6. Results of 100 ns MD simulation of the $A\beta_{16}$ System 5 from Table 1 ($C_{Zn} > C_{A\beta}$). Box size is 15 nm. The $A\beta_{16}$ molecules are shown with different colors. Zinc coordinating side chains are shown. Zinc ions are highlighted in blue.

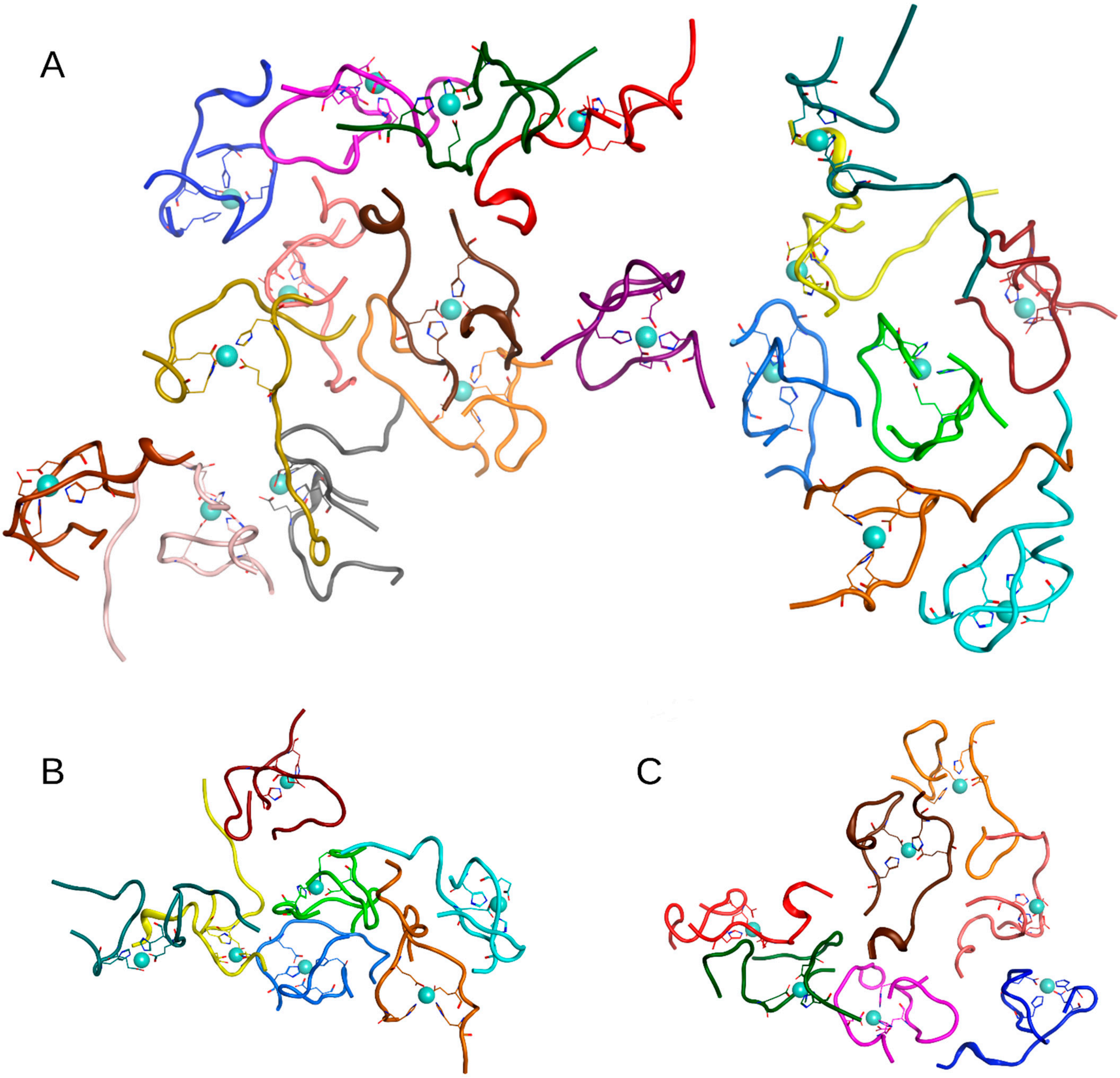

Figure S7. Results of 200 ns MD simulation of the Aβ16 System 2 from Table 1 ($C_{Zn} < C_{A\beta}$). Box size is 15 nm. The Aβ16 molecules are shown with different colors. Zinc coordinating side chains are shown. Zinc ions are highlighted in blue.

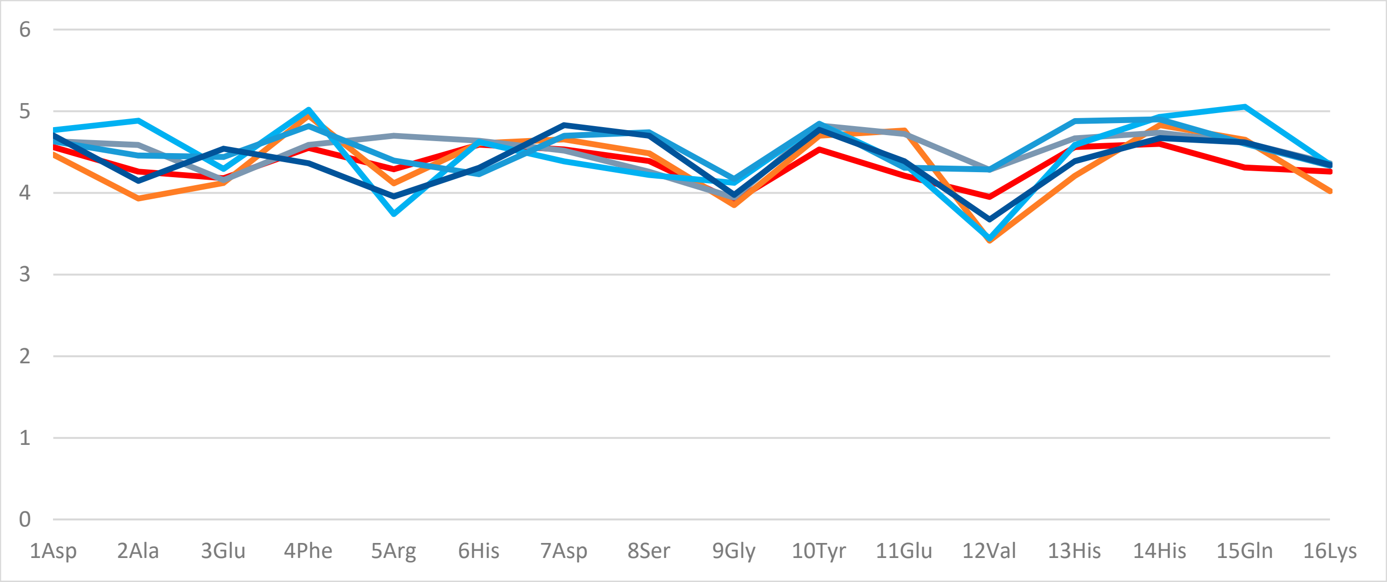

Figure S8. Chemical shifts (ppm) of the Hα atoms of isoD7-Aβ_{16}, measured with NMR in the presence of twofold molar excess of $ZnCl_2$ in 10 mM bis-Trid-d19 buffer, pH 6.8 (red line) in comparison with chemical shifts, calculated from the MD data: calculated with SPARTA+ server for PDB:2MGT structure (orange line) and for four peptide structures from the resulting quadromer structure from REMD simulation (blue lines). .
